# The Genetic Profile from HLA and Non-HLA Loci Allows Identification of Atypical Type 2 Diabetes Patients

**DOI:** 10.1155/2015/485132

**Published:** 2015-07-27

**Authors:** Matias Fabregat, Mariana Fernandez, Gerardo Javiel, Graciela Vitarella, Adriana Mimbacas

**Affiliations:** ^1^Biodiversity and Genetic Department, Instituto de Investigaciones Biológicas Clemente Estable, 11600 Montevideo, Uruguay; ^2^Teaching Care Unit, Unit of Diabetes “Hospital Pasteur”, ASSE, Ministry of Public Health, 11600 Montevideo, Uruguay; ^3^Diabetology Service of “Centro de Asistencia del Sindicato Médico del Uruguay”, 11800 Montevideo, Uruguay

## Abstract

The complex diagnosis and treatment of diabetes highlight the need for markers to define how to monitor patients correctly during the course of their disease. Different studies demonstrate the existence of patients who cannot be clearly classified. We have previously shown that it is possible to differentiate “atypical diabetic patients” based on genotyping the HLA. In this work we show that the analysis of non-HLA related to type 1 diabetes in the *INS-VNTR*, SNP rs689, and rs3842753 improves the identification of these patients. We genotyped 913 individuals comprising controls from the general population and “classic” and “atypical” diabetic patients. We compared the distribution of these loci and analyzed linkage disequilibrium. The haplotype was in LD for all the SNPs that were evaluated. Regarding their association with the disease, the haplotype IAC was associated with type 1 (odds 2.60, 1.82–3.72, CI 95%) and “atypical diabetes” (odds 1.50, 1.01–2.23, CI 95%), whereas we did not observe an association with type 2 diabetes. Therefore, our results confirm that atypical diabetes is a different entity of the disease where the patient presents with a genetic background of T1D and a T2D phenotype, findings that are likely to be relevant for patient diagnosis and management in the clinic.

## 1. Introduction

Uruguay is a little country located in South America with a population of 3.286.314 inhabitants according to the last census [1]. The population has been characterized as of European descent, with a small contribution of African descendants and native amerindian popultion [2]. Diabetes mellitus is a worldwide public health problem and Uruguay is not an exception showing a high prevalence of the disease, 8%, and an equal percent that are likely undiagnosed despite the small size of its population [3]. Diabetes is a multifactorial disease where genetic and environmental factors interact. This complexity could be an important influence in the actual classification and treatment of this pathology. Previously, in an effort to link the current classification with advances at molecular genetics we found that in several studies a significant proportion of diabetic patients did not present a correlation between genotype and phenotype [4–6]. Type 2 diabetes (T2D) diagnosed patients using international criteria [7] presented Human Leukocyte Antigen (HLA) alleles associated with type 1 diabetes (T1D) and positive antibodies in an intermediate value between both types, thus suggesting the coexistence of both types of diabetes. Several authors have published about this kind of diabetes [8–14] and these “atypical patients” cannot be grouped into any of the established groups by international guidelines. More importantly, these atypical patients do not have an adequate response when undergoing treatment [15].

Recently Maruthur and colleagues found evidence that in T2D the pharmacogenetic interactions for some antihyperglycemic drugs are consistent with their pharmacokinetics and pharmacodynamics, showing the importance of genetics variants [16]. In line with these observations we hypothesized that in “atypical patients” non-HLA genes, in addition to the HLA susceptibility alleles, could be influencing the lack of genotype-phenotype correlation and the course and development of the disease.

Here we perform an in depth analysis of the presence of other non-HLA loci related to T1D in this atypical diabetic population, particularly focusing on the insulin gene (Insulin-Varaibale number tandem repeat, SNP rs689, and rs3842753). We show that in the Uruguayan population these variants are in complete linkage disequilibrium [17]. This region is located on chromosome 11p15.5, in the promoter region of the insulin gene, and affects the level of transcription of* INS* and* IGF2* genes [10, 18]. Mathematical models estimate that, adding the* HLA* and* VNTR* contribution, up to 60% of the total genetic component of T1D is obtained [19].* VNTR* polymorphisms located in the insulin promoter, 596 bp upstream of the insulin start codon, consist of a highly polymorphic tandem repeat sequence ACAGGGGTGTGGGG (14 pb) whose variants are grouped into three classes of alleles according to their length [20, 21]. Regarding our population,* VNTR* is in linkage disequilibrium with rs689 where class I* VNTR* allele is associated with autoimmunity and inherited linked to the Adenine SNP allele. Furthermore the class III* VNTR* alleles are associated with autoimmunity protection and inherited linked to the Timina SNP allele. Population studies have shown that class III* VNTR* alleles have a dominant protective effect and are associated with a 60–70% reduction in the risk of developing T1D [19–23]. Even in the presence of autoantibodies and a high-risk HLA genotype, individuals carrying class III* VNTR* alleles present a significant reduction in the probability of developing the disease [24, 25]. Based on this association, major studies have used 23HphI (rs689) as an associated marker to study the* VNTR* [21, 26, 27].

Another SNP in linkage disequilibrium with the variants described above is rs3842753, a transversion at position +1140 relative to the initial translation codon of the* INS* gene. This polymorphism is located in the 3′UTR region of the* INS* mRNA and the cytosine-containing variant may cause instability of the mRNA [28]. In European and Uruguayan populations, the cytosine-containing allele is associated with T1D and is in complete linkage disequilibrium with the* VNTR* allele of class I and rs698 [17, 25]. Given the likely coexistence of both types of diabetes in our population and the presence of linkage disequilibrium as described in previous papers, we propose the in depth analysis of the genetic profile of these “atypical patients.” The analysis should include patients that present a phenotype characteristic of T2D with HLA susceptibility alleles associated with T1D compared to (a) the general population and (b) type 1 diabetes and type 2 diabetes patients without a HLA association.

## 2. Materials and Methods

In this unmatched case-control study, a total of 913 individuals, including diabetes patients (413) and controls (500), were enrolled between 2004 and 2012. Recruitment of patients was done by the outpatient health center of Montevideo city.

### 2.1. Control Samples

We selected 500 unrelated individuals from the DNA bank of the Department of Biodiversity and Genetics, IIBCE. This collection is a representative sample of Montevideo's general population and was randomly selected from 15 different medical institutions, public and private, when individuals assisted to their annual routine control.

Due to local legislation at the time (ref. 1081/1996), the MSP (Public Health Ministry) did not authorize collecting information about the clinical characteristics of patients when the study involved analyses of DNA samples. Therefore, the only characteristic recorded for this population is the age (>18 years old).

### 2.2. Diabetic Patient Samples

413 patients from 3rd attention level at Clinics for Diabetes of Reference Health Centers of Montevideo were analyzed. For the preparation of this study we only considered those patients receiving comprehensive care of their diabetes, following a nutritional plan and presenting a good adherence to physical activity according to their functional ability within the recommendations of the American Diabetes Association (ADA) and “Asociación Latinoamericana de Diabetes” (ALAD) [7, 29]. The diabetes samples were subclassified as type 1 diabetes, type 2 diabetes, and “atypical diabetes.”

#### 2.2.1. Type 1 Diabetes Patients (168)

Those patients were defined according to the ADA criteria [22], with an age of onset < 15 years and a body mass index < 25 kg/mts^2^.

#### 2.2.2. Type 2 Diabetes Patients, Classical and Atypical (153 + 92)

The population diagnosed with T2D was divided into two groups based on the presence or absence of T1D HLA susceptibility alleles described in the Uruguayan population [15, 30] according to the following inclusion criteria.

“Atypical diabetes” (92 individuals) (a) patients who had good adherence to the treatment; (b) they fulfilled the objectives of education and nutrition plans according to international guidelines; (c) present doubts on classification of diabetic type and/or not good therapeutic response (two consecutive measurements of glycated hemoglobin within three months not reduced in 1.5% [31]); (d) patients with susceptibility HLA alleles for autoimmune disease. We considered DQB1 ∗ 0201-0302 and DR 3-4 as susceptible ones in the Uruguay, (e) body mass index ≥ 25 kg/mts^2^ [32].

#### 2.2.3. Type 2 Diabetes (153 Individuals)

Those patients were fulfilling the same requirements a and b of atypical patients but without diagnostic doubts, responded to treatment, and do not present HLA alleles associated with autoimmune disease.

Samples from patients who had other endocrine disorders or tumors were excluded.

All subjects were interviewed by specialist medical doctors and gave a written informed consent to participate in the study. The protocol was approved by the Ethical Committee of the Ministry of Public Health (MSP) and the corresponding ethical committee of each participating institution.

### 2.3. Molecular Analysis

High molecular weight DNA extraction was performed from peripheral blood by standard phenol chloroform protocol.

The HLA typing was performed by reverse ASO technique (Innogenetics Ltd., Belgium, UE).* INS 5*′*VNTR*, rs3842753, and HLA were processed in a previous work [4, 5, 15, 17, 31].

The SNP rs689 (−23 HPh1) for controls and T2D were processed in a previous work [16, 33]: Atypical diabetes patients were genotyped by PCR-RFLP with the following primers: forward-5′AGCAGGTCTGTTCCAAGG-3′ and reverse-5′CTTGGGTGTGTAGAAGAAGC-3′ which amplifies a 360 bp fragment. The identification of the genotypes was performed by digesting the DNA fragments with* Bsm*AI. Sequencing was used for confirmation of genotypes (Macrogen, Ltda, Korea).

### 2.4. Statistical Analysis

(a) Calculations of power sample size were done with the Epi Info 3.4.3 database and the statistics software for public health professionals and the Quanto statistical package (http://biostats.usc.edu/software) considering the prevalence of diabetes in Uruguay (8%) [3]. We assumed 95% of confidential interval and power 80% in an unmatched case-control design.

(b) The statistical analysis of a polymorphism was done with the web tool for SNP analysis SNPStats (http://bioinfo.iconcologia.net/snpstats/start.htm).

(c) Selection of inheritance model: best inheritance mode was selected according to SNPStat program. The statistic follows a chi-square distribution with degrees of freedom equal to the number of additional parameters in the more complex model and Akaike information criterion (AIC) and Bayesian information criterion (BIC). The statistical test *P* values were calculated via an exact test. The reference category used by program was the homozygous form.

(d) Hardy-Weinberg equilibrium (HWE) for allelic and genotype frequencies was tested by chi-square test.

(e) Association between one polymorphism and disease: we make the contingency table and then apply a chi-square test and the estimation of the OR (odds ratio) for each genotype with respect to the reference genotype (Epi Info and SNPStat).

(f) Haplotype analysis: D statistic (under the assumption of no association) and correlation coefficient between alleles and the observed frequency were done. Linkage disequilibrium and haplotype were calculated with SNPStat program. Analysis of multiple SNPs and haplotype and analysis of association between haplotypes and disease were done.

## 3. Results 

Nine hundred and thirteen DNAs were analyzed in this study. Five hundred correspond to samples obtained from the general population of Uruguay and four hundred and thirteen correspond to diabetes patients. First we analyzed the clinical characteristics of diabetes patients and performed the comparison between the three groups defined previously (Table 1). We found a significant statistical difference when we compared T1D with any other subgroup in all variables considered. However, the only difference between T2D and “atypical diabetes” was in the level of HDL. These results are in accordance with previous reports [14]. Our genetic analysis showed that all SNPs analyzed in all groups were in Hardy-Weinberg equilibrium (HWE). The best heredity model for each SNP analyzed was the log-additive model, according to the AIC and BIC criteria of the SNPstat program. Allelic frequencies for all samples and association with the disease are shown in Tables 2(a), 2(b), and 2(c).

According to the* D*′ value and the correlation coefficient between alleles and the observed frequency, the evaluated haplotype was in linkage disequilibrium with all SNPs analyzed. The most frequent haplotype was IAC in controls and diabetes patients (Table 3). Haplotype with frequencies minor 1% was considered rare. Regarding the association with the disease, the high protective effect (IIITA) was present in T1D. The contingency table for haplotypes associated with T1D versus no presence of this haplotype revealed an association in T1D and “atypical diabetes” but resulted in no significant association in T2D (Table 4).

## 4. Discussion

Our study allowed us to analyze the genetic profile of “atypical diabetes” comparing the phenotype of type 2 diabetes with HLA susceptibility alleles in the general population, T1D, and T2D without HLA associated. Based on our results we propose that this marker can aid in the diagnosis and management of patients who present difficulties in control and follow-up. In addition, our analysis of the INS VNTR locus, as the second marker of importance for T1D susceptibility, further supports the notion that patients with difficulties in all approach areas (diagnosis, treatment, and evolution) have a strong genetic basis.

The last ADA guidelines classified diabetes in four clinical categories but recognized that some patients cannot be clearly classified as type 1 or 2 diabetes. Importantly, such difficulties in classification may occur at any age and genetic studies could improve the timely diagnosis in these kinds of patients, without having to wait for the disease to progress.

Mathematical models estimate that together the HLA and INS VNTR contribution may account for near 60% of the total genetic basis for developing T1D [19]. Therefore these markers, typically associated with autoimmune diabetes, when present in T2D patients, can modify the expected development of the pathology.

It has been shown that the second susceptibility locus for autoimmune disease, after HLA, corresponds to a mini satellite in the insulin gene (INS-VNTR) and in Caucasians populations this VNTR is in linkage disequilibrium with −23 HphI SNP. Previous studies estimate that 10% of the genetic susceptibility to T1D corresponds to this mini satellite [20].

In previous works, we showed that there are patients in our population who cannot be classified as either type 1 or type 2 diabetes according to the international guidelines. The clinical presentation, evolution, and difficulty in reaching expected therapeutic goals make them “atypical patients” in relation with HLA alleles [14]. Now we have gained insight into understanding the genetic basis of “atypical diabetes” by showing an association with genes different from HLA. It is noteworthy that, in these patients, there are no homozygous individuals for allele III of 5′*VNTR*. The protective allele III in the promoter region of* INS* is associated with a greater functional reserve of *β* cells in response to a direct hyperglycemic stimulus [34]. Although we did not analyze the functional reserve of beta cells, it is striking to find a high frequency of susceptibility allele I in “atypical patients” and lower presence of III alleles in their genotypes. This observation could suggest an earlier beta cell failure in these patients and therefore could be one possible explanation to understand the cellular basis of their bad treatment response, a possibility that should be analyzed in future investigations.

In conclusion, our results confirm that atypical diabetes is a different entity of the disease where patients combine a genetic background of T1D with a T2D phenotype, thus highlighting the importance of genetics as a new tool in clinical practice.

## Figures and Tables

**Table 1:** Clinical characteristics of diabetes patients.

Variable	Median ± SD			*P* corrected		
	DT1	DT2	AD	DT1 versus DT2	DT1 versus AD	DT2 versus AD
Age (years)	35.64 ± 17.4	65.05 ± 9.9	61.84 ± 13.4	<0.001	<0.01	0.05
BMI (Kg/m^2^)	22.96 ± 3.7	31.20 ± 5.7	30.74 ± 5.7	<0.01	<0.01	0.569
HbA1c (%)^∗^	9.85 ± 2.4	8.28 ± 1.7	8.32 ± 1.9	<0.01	<0.01	0.859
Cholesterol	4.88 ± 1.4	5.53 ± 1.1	5.23 ± 1.2	0.002	0.99	0.065
HDL	1.50 ± 0.5	12.67 ± 1.7	1.22 ± 0.3	0.013	0.001	0.041
LDL	2.80 ± 1.0	3.33 ± 1.7	3.01 ± 1.1	0.013	0.207	0.1
TG	1.28 ± 0.7	2.26 ± 1.4	2.21 ± 0.2	<0.001	<0.001	0.838

Units without bracket were expressed in IS, BMI = body mass index, TG = triglycerides, and HbA1c (%)^∗^ = glycated hemoglobin value at initial study.

**(a):** 

Variant	Allelic frequencies		OR	CI	*P* corrected
	Type 1 diabetes	Control			
INS-VNTR					
I	0.91	0.72	3.79	2.51–5.74	0.000
III	0.09	0.28			
rs689					
A	0.88	0.72	2.83	1.96–4.09	0.0000
T	0.12	0.28			
rs3842753					
A	0.86	0.72	2.44	1.72–3.47	0.0001
C	0.14	0.28			

**(b):** 

Variant	Allelic frequencies		OR	CI	*P* corrected
	Type 2 diabetes	Control			
INS-VNTR					
I	0.75	0.72	1.28	0.94–1.72	0.11
III	0.25	0.28			
rs689					
A	0.67	0.72	0.81	0.60–1.07	0.14
T	0.33	0.28			
rs3842753					
A	0.68	0.72	0.84	0.63–1.12	0.26
C	0.32	0.28			

**(c):** 

Variant	Allelic frequencies		OR	CI	*P* corrected
	Atypical diabetes	Control			
INS-VNTR					
I	0.82	0.72	1.75	1.13–2.71	0.01
III	0.18	0.28			
rs689					
A	0.79	0.72	1.51	1.51–2.25	0.04
T	0.21	0.28			
rs3842753					
A	0.74	0.72	1.23	0.84–1.79	0.31
C	0.24	0.28			

OR: odds ratio; CI: confidence intervals.

**Table 3:** Haplotype frequencies for all groups.

	INS	rs689	rs3842753	Freq.
Type 1 diabetes	I	A	C	0.7492
	III	T	A	0.2275
	I	T	A	0.012
	Rare	∗	∗	0.0113^∗^

Type 2 diabetes	I	A	C	0.7035
	III	T	A	0.2703
	I	T	A	0.0199
	Rare	∗	∗	0.0062

Atypical diabetes	I	A	C	0.7187
	III	T	A	0.2558
	I	T	A	0.0119
	^∗^Rare	∗	∗	0.0136

^∗^Means other haplotypes different to previous.

**Table 4:** Haplotype association with the disease.

	Haplotype	OR (95% CI)	*P* value
Diabetes type 1	IAC	2.60 (1.82–3.72)	<0.0001
	No IAC		

Diabetes type 2	IAC	0.83 (0.62–1.10)	NS
	No IAC		

Atypical diabetes	IAC	1.50 (1.01–2.23)	<0.05
	No IAC		

NS = non significant.
